# Neural Correlates of Executive Functioning in Anorexia Nervosa and Obsessive–Compulsive Disorder

**DOI:** 10.3389/fnhum.2022.841633

**Published:** 2022-05-26

**Authors:** Kai S. Thomas, Rosalind E. Birch, Catherine R. G. Jones, Ross E. Vanderwert

**Affiliations:** ^1^School of Psychology, Cardiff University, Cardiff, United Kingdom; ^2^Cardiff University Centre for Human Developmental Science, School of Psychology, Cardiff University, Cardiff, United Kingdom; ^3^Cardiff University Brain Research Imaging Centre, School of Psychology, Cardiff University, Cardiff, United Kingdom

**Keywords:** executive function, anorexia nervosa, obsessive–compulsive disorder, neuroimaging, transdiagnostic correlates

## Abstract

Anorexia nervosa (AN) and obsessive–compulsive disorder (OCD) are commonly reported to co-occur and present with overlapping symptomatology. Executive functioning difficulties have been implicated in both mental health conditions. However, studies directly comparing these functions in AN and OCD are extremely limited. This review provides a synthesis of behavioral and neuroimaging research examining executive functioning in AN and OCD to bridge this gap in knowledge. We outline the similarities and differences in behavioral and neuroimaging findings between AN and OCD, focusing on set shifting, working memory, response inhibition, and response monitoring. This review aims to facilitate understanding of transdiagnostic correlates of executive functioning and highlights important considerations for future research. We also discuss the importance of examining both behavioral and neural markers when studying transdiagnostic correlates of executive functions.

## Introduction

Anorexia nervosa (AN) and obsessive–compulsive disorder (OCD) are two mental health conditions that often co-occur (e.g., Serpell et al., 2002; Kaye et al., 2004; Meier et al., 2015) and have considerable genetic overlap (Lee et al., 2019; Yilmaz et al., 2020). In the updated DSM-5 (APA, 2013), OCD has been removed from its previous classification as an anxiety disorder and has subsequently been given its own category. It is characterized by obsessions which are heterogenous in nature, such as concerns surrounding pathological doubt, contamination and/or symmetry (Serpell et al., 2002). Compulsions are behaviors that serve to neutralize these obsessions and they include repetitive hand washing, checking, ordering, or mental acts such as repeating words or counting. These behaviors are often excessive, unrealistic, or irrational in proportion to the event, and can have a debilitating impact on the individual as a result (Serpell et al., 2002). AN is an eating disorder (ED) characterized by a distorted perception of body image that is linked to an intense fear of gaining weight, presented through extreme dietary restriction and weight loss (APA, 2013). Two AN subtypes have been identified: restricting type AN (AN-R) and binge/purge type AN (AN-BP). Individuals with AN-R display restrictive eating behaviors and/or excessive exercise, whilst those with AN-BP typically present with binge eating and/or purging behavior alongside restrictive eating behaviors (APA, 2013). A recent meta-analysis reported similar rates of co-occurring OCD (lifetime and current) in AN-R and AN-BP (Mandelli et al., 2020). Overall, in individuals with AN, 14% have a current co-occurring OCD diagnosis whilst 19% receive an OCD diagnosis across their lifetime (Mandelli et al., 2020). Prevalence rates of OCD in AN are considerably higher than rates in other ED diagnoses (Swinbourne et al., 2012; Mandelli et al., 2020), suggesting more similar mechanisms are at play in AN and OCD. OCD is considered a risk factor for AN (Bulik et al., 1997; Thornton and Russell, 1997; Buckner et al., 2010; Micali et al., 2011), and co-occurrence of these conditions is linked to poorer outcomes, such as an earlier age of ED onset, longer ED duration, and greater ED severity (Milos et al., 2002; Cumella et al., 2007).

Anorexia nervosa and OCD present with overlapping symptomatology, such as repetitive thoughts and preoccupations (e.g., AN: food, weight, body image etc.; OCD: contamination, symmetry etc.) alongside compensatory behaviors (e.g., AN: food restriction, compulsive exercise, binge/purge behaviors; OCD: hand washing, checking etc.) to reduce negative affect (Altman and Shankman, 2009). However, research aimed at understanding possible shared mechanisms that underlie this co-occurrence is limited. Executive functioning is a viable candidate for a shared mechanism as atypical profiles of executive functioning are seen in both AN and OCD (Gehring et al., 2000; Yano et al., 2016; King et al., 2019; Bora, 2020). Although executive dysfunction is considered a feature of many mental health and neurodevelopmental conditions (Hill, 2004; Castellanos et al., 2006; Nakano et al., 2008), AN and OCD have a number of shared traits, such as perfectionism, intolerance of uncertainty, restrictive cognitive style, and impulsivity (Altman and Shankman, 2009; Hoffman et al., 2012; Fourtounas and Thomas, 2016; Bohon et al., 2020). Shared executive functioning deficits could lead to these additional shared traits or symptomatology present in both disorders. On the other hand, there may be behavioral and neural indicators of executive functions that discriminate between the two disorders.

This review synthesizes research on executive functioning in AN and OCD, including their association with core clinical behaviors and broader trait profiles. To date, only two studies have directly compared these cognitive functions in AN and OCD (set shifting: Bohon et al., 2020; and response inhibition: Boisseau et al., 2012), reflecting the very limited consideration of the potential overlap in executive function. The review will therefore provide the first detailed exploration of shared and unique neural and behavioral correlates of these two disorders. Further, it will highlight opportunities for future research and development within this relatively under-researched area.

## Executive Functioning

Executive functions are a diverse set of cognitive skills involved in a broad range of activities, such as behavioral control, memory, planning, and cognitive flexibility (Thakkar et al., 2008; Diamond, 2013). Among studies that report impairments in executive functioning in individuals with AN and OCD, the components most widely studied are working memory, set shifting, response inhibition, and monitoring (e.g., AN: Tchanturia et al., 2002; Yano et al., 2016; Brooks et al., 2017; OCD: Endrass et al., 2008, 2010; Cavedini et al., 2010; Abramovitch et al., 2013; Kashyap et al., 2013; Snyder et al., 2015). Additionally, set shifting and response inhibition abilities are two executive functions that have been directly compared in AN and OCD samples (Boisseau et al., 2012; Bohon et al., 2020). Table 1 below provides brief definitions of these functions, as well as common tasks used to measure these in AN and OCD.

**TABLE 1:** Executive function processes and measures in Obsessive–Compulsive Disorder (OCD) and Anorexia Nervosa (AN) samples.

Function	Definition	Measures	Measures used in OCD and AN
Set shifting	Flexibly shifting attention between multiple tasks, behaviors, or mental states.	**Wisconsin card sorting task (WCST; Berg, 1948):** involves learning from feedback to sort cards according to one dimension (e.g., shape) and then shifting to a different dimension (e.g., color) when provided with negative feedback on the first dimension. **Trail Making Test (TMT; Heaton and Staff, 1993):** alternate/shift between connecting letters and numbers in sequence (e.g., A-1-B-2). **Intradimensional/extradimensional shift (ID/ED; e.g., CANTAB):** involves learning from feedback to select a stimulus based on one dimension, then performing an intradimensional shift to a previously non-reward stimulus. Then, an extradimensional shift to a different dimension. **Berg’s card sorting task (BCST; Berg, 1948):** follows the same procedure as the WCST. **Haptic Illusion task (Tchanturia et al., 2002):** measure of perceptual set shifting. During a habituation phase, participants judge the relative size of two different-sized balls with their eyes closed. Then, two same-sized balls are presented. Outcome is the number of perceptual illusions (same-sized balls perceived as different sizes). **Cat Bat Task (Tchanturia et al., 2002):** measure of cognitive set shifting. Involves completing a word with missing letters in two different contexts (either completing “cat” or “bat”). Requires shift in cognitive set. **Visual Target-Detection task (e.g., Zastrow et al., 2009)**: involves categorizing stimuli (geometric shapes) as target, non-target or standard stimuli. Non-target and standard stimuli required one response, whilst target stimuli required an alternative response. Target stimuli were defined at the start of each set and changed after every two sets, resulting in conditions where the target changed at the start of each set (shift – behavioral and cognitive set shifting) or stayed the same (maintain – behavioral set shifting).	OCD **WCST** (e.g., Lawrence et al., 2006; Andrés et al., 2007; Bohon et al., 2020), **TMT** (e.g., Aronowitz et al., 1994; Schmidtke et al., 1998; Andrés et al., 2007), **ID/ED** (e.g., Chamberlain et al., 2007; Hybel et al., 2017a) AN **WCST** (e.g., Galimberti et al., 2013; Sato et al., 2013; Castro-Fornieles et al., 2019), **TMT** (e.g., Holliday et al., 2005; Tenconi et al., 2010; Herbrich et al., 2018), **BCST** (e.g., Danner et al., 2012; Lindner et al., 2014), **Haptic Illusion task** (e.g., Tchanturia et al., 2002, 2004a; Holliday et al., 2005), **Cat Bat task** (e.g., Tchanturia et al., 2002, 2004a; Holliday et al., 2005), **Visual Target-Detection task** (e.g., Zastrow et al., 2009).
Working memory	Actively maintaining information “on line” across a short delay.	**Digit Span (backward or forward versions; as part of the WAIS-IV or WISC-V)**: repeat a sequence of numbers in reverse or forward order. **Verbal *n*-back (e.g., Koch et al., 2012)**: specify if the stimulus (usually a letter) matches the stimulus *n* (e.g., 1, 2, or 3) items back. **Spatial *n*-back (e.g., van der Wee et al., 2003)**: specify if the stimulus location matches the location *n* (e.g., 1, 2, or 3) items back. **Arithmetic test (as part of the WAIS-IV or WISC-V):** solving mental arithmetic problems. **Spatial working memory test (SWM; e.g., CANTAB):** presented with a number of colored squares/boxes and through a process of elimination, the participant must try and find a number of “tokens” in each of a number of boxes. **Spatial span (backward or forward versions; e.g., CANTAB):** click irregularly arranged squares in the opposite/same order as they light up on the computer screen. **Letter-number sequencing (LNS; as part of the WAIS-IV or WISC-V):** recall a string of letters and digits by repeating numbers in chronological order, followed by letters in alphabetical order.	OCD **Digit span** (e.g., Ozcan et al., 2016; Bernardes et al., 2020), **Arithmetic test** (e.g., Abramovitch et al., 2015; Geller et al., 2018), ***n-*back** (e.g., Ornstein et al., 2010; Koch et al., 2012; de Vries et al., 2014), **SWM** (e.g., Hybel et al., 2017a,b), **Spatial span** (e.g., Ornstein et al., 2010; Hybel et al., 2017a,b) AN **Digit span** (e.g., Bentz et al., 2017; Zegarra-Valdivia and Chino-Vilca, 2018), ***n-*back test** (e.g., Dickson et al., 2008; Lao-Kaim et al., 2014; Israel et al., 2015), **SWM test** (e.g., Bentz et al., 2017), **Spatial span** (e.g., Phillipou et al., 2015; Bentz et al., 2017), **LNS** (e.g., Biezonski et al., 2016; Bentz et al., 2017)
Response inhibition	Withhold a prepotent response.	**Go/No-Go (e.g., Bohne et al., 2008):** based on a cue, quickly categorize and respond to some stimuli but withhold a response to other stimuli. **Stop-signal task (SST; see Lipszyc and Schachar, 2010, for a review):** quickly categorize and respond to stimuli, unless a stop signal appears, signaling to withhold a response.	OCD **Go/No-Go** (e.g., Bannon et al., 2002; Herrmann et al., 2003; Kim et al., 2007; Roth et al., 2007; Bohne et al., 2008; Tolin et al., 2014), **SST** (e.g., Menzies et al., 2007; Boisseau et al., 2012; de Wit et al., 2012; Lei et al., 2015; Fan et al., 2017) AN **Go/No-Go** (e.g., Lock et al., 2011; Suttkus et al., 2021), **SST** (e.g., Oberndorfer et al., 2011; Galimberti et al., 2012; Wierenga et al., 2014; Collantoni et al., 2016)
Response monitoring	Monitoring responses and their outcomes and adapting behavior to optimize performance.	**Flanker task (Eriksen and Eriksen, 1974):** typically arrows or letters are presented with congruent (>>>>>) or incongruent (>><>>) conditions. Participants must respond to the middle stimuli by pressing one of two buttons (mapped onto arrow direction) as quickly and accurately as possible. **Go/No-Go:** as described above. **Stroop task (Stroop, 1935):** requires inhibition of cognitive interference, as the processing of a stimulus attribute (e.g., color) then interferes with the simultaneous processing of another feature (e.g., word/name) of the same stimulus. **Probabilistic Reversal Learning task (PRL; Hampton et al., 2006):** required to learn and respond to stimulus-reward contingencies and monitor covert changes in these and subsequently adapt behavior. Also includes probabilistic feedback (receive negative feedback despite correct response and vice versa). **Stop-signal task (SST):** as described above.	OCD **Flanker** (e.g., Endrass et al., 2008; Riesel et al., 2011), **Go/No-Go** (e.g., Ruchsow et al., 2005), **Stroop** (e.g., Gehring et al., 2000; Carmi et al., 2019), **PRL** (e.g., Nieuwenhuis et al., 2005) AN **Flanker** (e.g., Pieters et al., 2007), **Go/No-Go** (e.g., Suttkus et al., 2021), **SST** (e.g., Wierenga et al., 2014), **PRL** (e.g., Geisler et al., 2017; Ritschel et al., 2017)

*WAIS-IV, Wechsler Adult Intelligence Scale (4th edition; Wechsler, 2008); WISC-V, Wechsler Intelligence Scale for Children (5th edition; Wechsler, 2014); CANTAB, Cambridge Neuropsychological Test Automated Battery (CANTAB Eclipse, 2006).*

A narrative review was chosen to synthesize the broad range of literature covering executive functioning in AN and OCD, as well as the limited direct comparison between the two disorders. Thorough reviews of the literature were conducted using Web of Science and PubMed databases. Search terms were combinations of “Anorexia” and “OCD” with “Executive function*,” “Set shifting,” “Inhibition,” “Memory,” and “Monitoring.” In order to specifically examine difference between adults and children, these terms were further specified in searches e.g., “Set shifting, Anorexia, Children.” Research articles were screened for relevance and focused on individuals with a diagnosis of AN or OCD; literature examining subclinical symptomatology was excluded. We expanded our search by following up relevant citations, e.g., from systematic reviews and meta-analyses, resulting in our search snowballing and reaching saturation.

### Set Shifting

Set shifting is defined as the ability to flexibly shift attention between multiple tasks, behaviors, or mental states (Miyake et al., 2000). Set shifting impairments have been frequently reported in adults with OCD compared to healthy controls (HCs; e.g., Aronowitz et al., 1994; Veale et al., 1996; Okasha et al., 2000; Watkins et al., 2005; Lawrence et al., 2006; Bora, 2020). Findings from meta-analyses indicate these impairments are moderate, with effect sizes around *d* = 0.5 (Abramovitch et al., 2013; Shin et al., 2014; Snyder et al., 2015). These impairments remain stable across time, with similar difficulties observed in individuals with symptomatic OCD and individuals who are asymptomatic (i.e., post-treatment; Bannon et al., 2006). In contrast, set shifting performance has been reported to improve following cognitive behavioral therapy (CBT) in individuals with OCD (Kuelz et al., 2006; Voderholzer et al., 2013), suggesting set shifting impairments may not be a trait of the disorder. There is evidence that set shifting difficulties may represent a biomarker of subthreshold OCD (Chamberlain et al., 2007; Rajender et al., 2011; Bora, 2020); although, this appears to be dependent on the task and outcome measures studied (Abramovitch et al., 2021).

Set shifting difficulties have also been reported in children with OCD compared to HCs (e.g., Andrés et al., 2007; Shin et al., 2008); however, more recent studies contradict these findings, as they did not find significant associations between OCD symptoms and set shifting performance (e.g., Hybel et al., 2017a; Geller et al., 2018). It is worth noting that although there is some evidence for poorer set shifting performance in children with OCD compared to HCs, this difference in performance may not be clinically meaningful (for more details, see Abramovitch et al., 2013) as effect sizes are relatively small (e.g., Abramovitch et al., 2015). In the only study to-date that has directly compared set shifting performance in AN and OCD, adolescents with OCD demonstrate impaired set shifting in comparison to both HCs and weight-restored adolescents with AN (Bohon et al., 2020). This suggests that in adolescents, behavioral markers of set shifting deficits are more characteristic of OCD than AN.

In AN, set shifting difficulties have been observed in those in the acute phase of AN (Tchanturia et al., 2002, 2004a) and in recovery (Roberts et al., 2010; Danner et al., 2012), as well as in unaffected family members (Holliday et al., 2005; Tenconi et al., 2010; Galimberti et al., 2013) compared to HCs. Effect sizes from meta-analyses suggest set shifting impairments are moderate when comparing adults with AN and HCs (e.g., Wu et al., 2014; Westwood et al., 2016). Even though set shifting difficulties have been proposed to be a risk factor for AN (Holliday et al., 2005; Lindner et al., 2014), most studies in children and adolescents with AN have found no significant impairments in set shifting compared to HCs (Bühren et al., 2012; Lang et al., 2014; Rößner et al., 2016; Herbrich et al., 2018). Although set shifting difficulties may be more characteristic of OCD than AN in adolescents (e.g., Bohon et al., 2020), findings from both disorders suggest set shifting difficulties are less common in childhood and adolescence than in adulthood. These differences may be dependent on the set shifting task used across the age span. For example, a meta-analysis focused on performance in the Wisconsin Card Sorting Task (WCST) found greater effect sizes for set shifting impairments in adults with AN compared to those in children and adolescents with AN (Westwood et al., 2016). However, in another meta-analysis, comparable effect sizes between adolescents and adults with AN were found across a range of set shifting tasks (Wu et al., 2014). Alternatively, compensatory mechanisms may mask subtle difficulties present in younger age groups. Weinbach et al. (2019) reported that compared to HCs, adolescents with weight-restored AN displayed enhanced performance on set shifting tasks assessed using the Delis–Kaplan Executive Function System (DKEFS; Delis et al., 2001). However, once inhibition ability was controlled for, poorer set shifting performance was found in adolescents with weight restored AN compared to HCs (Weinbach et al., 2019). These findings suggest that in adolescence, inhibition performance could compensate for difficulties in set shifting on tasks that require both inhibition and set shifting, leading to null findings.

In regard to AN subtypes, some studies in adults and adolescents report no differences in set shifting ability between AN-R and AN-BP (e.g., Van Autreve et al., 2013; Van Autreve and Vervaet, 2015). In contrast to the set shifting impairments discussed so far, one study has reported superior set shifting performance in AN compared to HCs. Herbrich et al. (2018) found adolescents with AN-R displayed faster responses on the Trail Making Test (TMT) compared to HCs, which indicates superior set shifting, whilst there was no significant difference between AN-BP and HCs. The authors suggested this differential finding could be explained by the higher levels of perfectionism often seen in individuals with AN-R (e.g., Nilsson et al., 2008). In contrast to the deficits in set shifting performance commonly reported, increased personal standards perfectionism, thought to be an adaptive dimension of perfectionism, has been associated with enhanced set shifting performance in both individuals with a current AN diagnosis (Vall and Wade, 2015) and individuals who have recovered from AN (Lindner et al., 2014). This demonstrates an important contradiction as increased perfectionism is commonly reported in both AN and OCD (e.g., Summerfeldt et al., 2004; Barke et al., 2017). In contrast to the findings in AN, there is no clear association between set shifting performance and perfectionism in OCD. This relation between superior set shifting performance and perfectionism may be specific to AN-R, as enhanced performance has been reported in this group (described above; e.g., Herbrich et al., 2018). Alternatively, or in addition, this relation may be specific to the adaptive dimension of perfectionism rather than the maladaptive dimension (socially prescribed perfectionism) or a global measure of perfectionism. Taken together, these findings suggest difficulties in set shifting performance may be more characteristic of individuals with AN-BP. Those with AN-R may exhibit enhanced set shifting performance due to increased personal standards perfectionism. Previous null findings from studies comparing set shifting performance in individuals with AN and HCs may be explained by over-sampling of individuals with AN-R compared to AN-BP. However, there is still a clear need for further research to distinguish between AN subtypes when examining set shifting performance, as well as controlling for both adaptive and maladaptive dimensions of perfectionism.

Impairments in set shifting are thought to be related to a more rigid, rule-bound, and less adaptive cognitive style, which is observed in both AN and OCD when compared to HCs (Tchanturia et al., 2004b; Meiran et al., 2011; King et al., 2019; Bohon et al., 2020; Heinzel et al., 2021). Set shifting impairments have also been associated with more severe ED rituals (Roberts et al., 2010), and childhood rigidity and inflexibility (Tchanturia et al., 2004a). This cognitive style could also contribute to the maintenance of AN through more rigid and compulsive behaviors resulting in chronic and severe forms of the illness (Harrison et al., 2011). Set shifting impairments have also been reported in community samples who display checking behavior, which is a hallmark of OCD and a behavior commonly seen in AN, specifically related to body checking (e.g., Calugi et al., 2017). Taken together, the behavioral evidence suggests that impaired set shifting may perpetuate intrusive compulsive and repetitive behaviors by preventing the individual from shifting their attention away; manifesting in checking (OCD) and purging (AN-BP) behaviors. Conversely, in AN-R, enhanced set shifting performance may enable the individual to better shift attention away from food and facilitate restrictive eating behaviors.

In adults with OCD, set shifting difficulties are associated with reduced fronto-striatal activation when compared to HCs (e.g., Morein-Zamir et al., 2016; Vaghi et al., 2017). This can result in an imbalance in brain activation between fronto-striatal circuits, with hypoactivation of the dorsal fronto-striatal regions, such as the dorsolateral prefrontal cortex (PFC) and the dorsal anterior cingulate cortex (dACC), and increased activation in ventral fronto-striatal regions, such as the ventromedial PFC and orbitofrontal cortex (OFC), during set shifting (Gu et al., 2008). These findings suggest individuals with OCD are placing more value on repeated tasks or have difficulty shifting from the previous set. Relating to difficulties shifting set, hyperactivation of both the inferior and middle frontal gyrus has also been associated with perseverative errors in adolescents with OCD compared to HCs (Bohon et al., 2020). In conjunction with the PFC, the basal ganglia has been implicated in set shifting in healthy populations (Monchi et al., 2006). In both OCD probands and asymptomatic first-degree relatives, structural abnormalities within the putamen have been associated with set shifting errors, suggesting structural changes to the putamen linked to poor cognitive flexibility may confer familial risk for OCD (Isobe et al., 2021).

In AN, hypoactivation of fronto-striatal regions has also been implicated in set shifting difficulties compared to HCs (see Table 2 for a summary of the comparisons between AN and OCD; Zastrow et al., 2009; Sato et al., 2013; Lao-Kaim et al., 2015). For example, hypoactivation of the thalamus, ventral striatum, ACC, sensorimotor brain regions, and the cerebellum has been found in adults with AN compared to HCs during behavioral response shifting, but not cognitive set shifting (Zastrow et al., 2009). However, individuals with AN have also been found to display dominant activation in frontal and parietal regions, including the middle frontal gyrus, temporoparietal junction and precuneus, during behavioral shifting compared to HCs (Zastrow et al., 2009; Lao-Kaim et al., 2015). This is thought to reflect increased supervisory cognitive control during set shifting task performance in AN (Zastrow et al., 2009). Hyperactivation of frontal and parietal regions during visual stimulus processing has also been associated with better set shifting ability in individuals with AN but not HCs (Sultson et al., 2016), and may help explain the persevered behavioral performance that is sometimes seen in individuals with AN compared to HCs (e.g., Zastrow et al., 2009; Lao-Kaim et al., 2015). In contrast to Zastrow et al.’s (2009) findings, some studies have failed to find different patterns of activation in the ACC between individuals with AN and HCs (Sato et al., 2013; Castro-Fornieles et al., 2019). This may be due to differences in tests used to probe set shifting ability (see Table 1 for task descriptions), as well as the discrepancies between the types of set shifting being measured (e.g., behavioral vs. cognitive set shifting). For example, Zastrow et al. (2009) used a target-detection set shifting task that required a behavioral response shift when a new overt behavioral strategy was presented; whereas, the WCST involves a covert rule change. Using the WCST, Sato et al. (2013) reported hypoactivation of the ventrolateral PFC and parahippocampal cortex in adults with AN compared to HCs.

**TABLE 2:** Summary of the comparison between Anorexia Nervosa (AN) and Obsessive–Compulsive Disorder (OCD) in each examined executive function.

	Set shifting	Working memory	Response inhibition	Response monitoring
Behavioral	Impairments consistently reported in adults with AN and OCD during the acute/symptomatic phase (e.g., Tchanturia et al., 2002, 2004a; Lawrence et al., 2006; Bora, 2020) and in the recovery/asymptomatic phase (e.g., Bannon et al., 2006; Roberts et al., 2010; Danner et al., 2012). In children and adolescents, impairments appear more characteristic of OCD than AN (e.g., Bohon et al., 2020).	Visuospatial working memory deficits in both disorders (e.g., Kemps et al., 2006; Shin et al., 2014). Preoccupying thoughts and ruminations may provide a link between working memory impairments in both AN and OCD (e.g., Harkin and Kessler, 2009; Brooks et al., 2012). Some evidence of verbal working memory deficits in AN (Biezonski et al., 2016; Zegarra-Valdivia and Chino-Vilca, 2018) but only in OCD studies with a greater proportion of females (Snyder et al., 2015). More reliable findings of working memory impairments in children and adolescents with OCD compared to those with AN (Castro-Fornieles et al., 2010; Diwadkar et al., 2015).	Impairments consistently reported in adults with OCD and AN-BP subtype (e.g., de Wit et al., 2012; Galimberti et al., 2012; Lei et al., 2015; Collantoni et al., 2016). These impairments may lead to higher levels of impulsivity seen in both OCD and AN-BP subtype (e.g., Altman and Shankman, 2009; Hoffman et al., 2012). Enhanced performance is reported in adolescents with AN-R subtype (Weinbach et al., 2020).	Majority of studies report no differences between healthy controls and AN (Wierenga et al., 2014; Geisler et al., 2017; Suttkus et al., 2021) and OCD (Riesel et al., 2011; Hanna et al., 2012; Carrasco et al., 2013).
Neurological	Hypoactivation in fronto-striatal circuits in adults with AN (Zastrow et al., 2009; Sato et al., 2013; Lao-Kaim et al., 2015) and OCD (Morein-Zamir et al., 2016; Vaghi et al., 2017). Some evidence of hyperactivation of frontal areas, such as the middle frontal gyrus, in both disorders (e.g., Zastrow et al., 2009; Bohon et al., 2020).	Hyperactivation of parietal regions is observed in both children and adolescents with AN and OCD (Castro-Fornieles et al., 2010; Diwadkar et al., 2015). Hyperactivation of the dorsolateral PFC occurs alongside preserved or enhanced performance in both disorders (Ciesielski et al., 2005; Henseler et al., 2008; Brooks et al., 2014; Israel et al., 2015).	Fronto-striatal circuitry is implicated in both AN (e.g., Lock et al., 2011; Wierenga et al., 2014; Bartholdy et al., 2019) and OCD (e.g., Roth et al., 2007; Kang et al., 2013). Dorsolateral PFC activity is implicated in OCD (e.g., Woolley et al., 2008; Page et al., 2009) and binge/purging eating behaviors (Lock et al., 2011). Hypoactivation of the frontal gyrus reported in both disorders (e.g., de Wit et al., 2012; Collantoni et al., 2016). Hypoactivation of the dorsolateral PFC in OCD (e.g., Woolley et al., 2008; Page et al., 2009) whilst hyperactivation is found for those with binge/purging eating behaviors (Lock et al., 2011). Binge/purge behaviors in adolescents associated with greater recruitment of medial PFC and ACC (Bartholdy et al., 2019), whilst in OCD there is hypoactivation of the medial PFC and ACC (Norman et al., 2016).	In OCD, enhanced ERN amplitudes and elevated ACC activity are consistently reported in adults (Johannes et al., 2001; Nieuwenhuis et al., 2005; Endrass et al., 2008, 2010; Stern et al., 2010; Riesel et al., 2011) and children and adolescents with a diagnosis of OCD (Huyser et al., 2011; Hanna et al., 2012; Carrasco et al., 2013). Whilst in AN, attenuated ERN amplitudes have been reported (Pieters et al., 2007).

*PFC, prefrontal cortex; ACC, anterior cingulate cortex; AN-BP, anorexia nervosa – binge/purge; AN-R, anorexia nervosa – restricting; ERN, error-related negativity.*

When comparing set shifting performance between individuals with AN and OCD, it is worth highlighting the variability in studies. The vast majority of studies examining set shifting performance in AN are with females; whereas in OCD samples, there is a more equal distribution of males and females. This is important as previous research has reported gender to be a significant moderator of set shifting performance in individuals with OCD, such that samples with more female participants had worse performance (Snyder et al., 2015). Therefore, there may be an important role of gender in set shifting ability within these disorders, which has not adequately been explored. Further, most studies examining set shifting ability in AN and OCD use the WCST and/or TMT (see Table 1). However, some tasks used to capture specific types of set shifting in AN, such as the Haptic Illusion task used to measure perceptual set shifting (e.g., Tchanturia et al., 2002), have not been used in both disorders. In addition, task-specific differences, such as variation in novelty and introduction of feedback, have been found to influence striatal activity (Rauch et al., 1997; Monchi et al., 2001). Finally, sampling for neuroimaging studies has not differentiated between AN-R and AN-BP subtypes. For example, it is possible that the hypoactivation of the fronto-striatal regions in both OCD and AN may reflect the overlap in set shifting impairments between OCD and AN-BP. This adds to the complexity of comparing previous findings from AN and OCD samples across varying tasks but offers important future directions for research in this area.

Overall, set shifting has been proposed as a trait marker for both AN and OCD and is thought to be linked to a more restrictive and less adaptive cognitive style. Findings from both disorders suggest dysfunction of fronto-striatal neurocircuits are central to set shifting difficulties. However, the specific brain regions and behavioral results reported in previous studies are inconsistent. This may reflect the variety of tasks used to measure set shifting ability and the specific outcome measures used, as well as the additional executive functions often required in these complex tasks.

### Working Memory

Working memory involves the ability to actively maintain and manipulate information “on-line” across a short delay (Baddeley, 1992). Three independent meta-analyses have found significant working memory impairments with small to moderate effect sizes for adults with OCD compared to HCs (Abramovitch et al., 2013; Shin et al., 2014; Snyder et al., 2015). Several researchers have drawn links between working memory deficits and obsessive-compulsive behaviors, such as checking behaviors, obsessions, and ruminations (e.g., Harkin and Kessler, 2009; Jaafari et al., 2013). These behaviors have been proposed to impede the capacity of cognitive effort for manipulating and processing working memory content (Jaafari et al., 2013). Overall, working memory impairments appear more pronounced for visuospatial working memory, compared to verbal working memory in OCD (Shin et al., 2014; Snyder et al., 2015). However, studies with a greater proportion of females are associated with larger impairments in verbal working memory (Snyder et al., 2015). This warrants further investigation as previous research has identified reduced performance in verbal fluency tasks, as well as the Wechsler Adult Intelligence Scale-Revised (WAIS-R) Digit Span-forward test in women with OCD compared to healthy women (Mataix-Cols et al., 2006). There is evidence that working memory impairments may be a biomarker of OCD, as both adult and child first-degree relatives show comparable working memory deficits to OCD probands (Ozcan et al., 2016; Zartaloudi et al., 2019; Bernardes et al., 2020). Again, these difficulties appear to be more pronounced in visuospatial working memory and verbal fluency, rather than a broader working memory deficit. Studies examining outcomes of pharmacological therapies provide further support for trait-like impairments, as individuals with OCD display persistent visuospatial working memory deficits 3–4 months after starting selective serotonin reuptake inhibitor (SSRI) treatment (Kim et al., 2002; Nielen and Den Boer, 2003). However, it is worth highlighting that some individuals were still symptomatic at follow up (Kim et al., 2002), suggesting further long-term follow-up studies are needed to draw strong conclusions of trait-like impairments in visuospatial working memory.

Assessment of working memory performance in children with OCD is mixed. Many large studies report no differences to HCs in working memory performance (Ornstein et al., 2010; Hybel et al., 2017b) and a meta-analysis found small effect sizes for impaired working memory in children and adolescents with OCD, none of which reached statistical significance (Abramovitch et al., 2015). This distinction between adult and child populations may reflect method factors as the research into children with OCD has typically relied on working memory tasks from the Wechsler Intelligence Scale for Children (WISC). The digit span test is a measure of verbal working memory and both adults and children with OCD display proficient performance on this task (e.g., Andrés et al., 2007; Shin et al., 2014; Ozcan et al., 2016; Geller et al., 2018). Studies employing alternative methods of working memory assessment in children with OCD are more likely to identify impairments (Marzuki et al., 2020). For example, impairments were reported on the Finger Windows Test (Chang et al., 2007) and a task-switching paradigm (Wolff et al., 2017) compared to HCs. This may reflect an overall trend toward impaired visuospatial working memory performance in both adults and children with OCD, whilst verbal working memory remains intact. However, it is also worth considering that visuospatial working memory tasks traditionally vary more in complexity and task load, compared to verbal working memory tasks (Abramovitch and Cooperman, 2015). Abramovitch and Cooperman (2015) present a pattern across studies whereby more complex and difficult tasks assessing both visuospatial and verbal working memory produce comparable impairments in individuals with OCD.

Similar to OCD, the pattern of findings across the lifespan in AN suggests impairments in working memory performance compared to HCs (see Table 2 for a summary of comparisons between AN and OCD). In AN, these impairments have been framed as preoccupying cognitions interfering with working memory performance (Kemps et al., 2006; Brooks et al., 2012). A recent review by Brooks et al. (2017) report that, overall, individuals with AN either display poorer working memory performance or no difference in performance compared to HCs (Phillipou et al., 2015; Biezonski et al., 2016; Bentz et al., 2017; Giombini et al., 2017; Ritschel et al., 2017; Solstrand Dahlberg et al., 2017). In parallel to OCD, some studies suggest a more pronounced visuospatial working memory impairment in AN compared to HCs (e.g., Kemps et al., 2006), specifically during high cognitive demand (Phillipou et al., 2015). However, in contrast to OCD, deficits in verbal working memory have been commonly identified in AN compared to HCs (e.g., Biezonski et al., 2016; Zegarra-Valdivia and Chino-Vilca, 2018). A previous review by Brooks (2016) has identified several covariates that may affect the patterns of findings, and which are often different across studies, such as duration of illness (Dickson et al., 2008; Lao-Kaim et al., 2014), age, body mass index (BMI), and education (Israel et al., 2015). The heterogeneity of AN itself is also important to consider, as AN-BP symptoms are associated with worse working memory performance, whereas AN-R symptoms are associated with better working memory performance (Israel et al., 2015). For example, superior working memory performance has been reported in female adolescents with AN (Hatch et al., 2010) and female adults with AN-R (Dickson et al., 2008) compared to HCs in variations of the n-back task. These findings highlight the need for future research to examine working memory performance in both AN subtypes compared to HCs.

Neuroimaging studies examining correlates of working memory processes have identified involvement of the dACC, dorsal PFC, and parietal cortex in healthy adults and children (Diwadkar et al., 2015; Yaple and Arsalidou, 2018; Yaple et al., 2019). In children with OCD, there is hyperactivation of frontal and parietal regions, such as the dorsolateral PFC, parietal lobe, and middle frontal gyrus, compared to HCs (Diwadkar et al., 2015). This activity is modulated by dACC activity during both high and low working memory demands and proposed to reflect inefficient control-related networks in children with OCD (Diwadkar et al., 2015). In adolescents with AN, hyperactivity is similarly reported in parietal regions, and also in the temporal gyrus, during a working memory task compared to HCs (Castro-Fornieles et al., 2010). Interestingly, in this study there were no behavioral differences between these two groups, suggesting that the AN group had increased cognitive effort in order to perform similarly to HCs. Importantly, the differences in fMRI response reported by Castro-Fornieles et al. (2010) disappeared following weight-restoration, possibly due to functional brain improvement associated with weight recovery. It is worth highlighting that although studies in both OCD and AN samples employed an n-back task, the working memory load varied in adolescents with OCD e.g., maintaining 0, 1, or 2 items in memory (Diwadkar et al., 2015), but remained constant in adolescents with AN, e.g., only maintaining 1 item in memory (Castro-Fornieles et al., 2010). In order to improve direct comparisons between OCD and AN, future research should employ n-back tasks with varying working memory loads. This is especially important given the involvement of the dACC across different working memory loads reported in OCD samples and the absence of dACC activity reported in AN studies.

Findings from adults with OCD appear to reflect the pattern observed in children. General hyperactivation is reported in frontal and parietal regions, including the dorsolateral PFC, superior temporal gyrus (Ciesielski et al., 2005; Henseler et al., 2008; Nakao et al., 2009), ACC (e.g., van der Wee et al., 2003; Henseler et al., 2008), and insula (Ciesielski et al., 2005; Nakao et al., 2009), compared to HCs. This hyperactivation is alongside preserved behavioral performance in some of these studies (Ciesielski et al., 2005; Henseler et al., 2008), and may therefore reflect compensatory working memory mechanisms (Ciesielski et al., 2005; Henseler et al., 2008; de Vries et al., 2014). Findings from studies that manipulate task difficulty (e.g., increased or decreased *n* in an n-back task) have produced contrasting findings. For example, at higher cognitive loads, studies report hypoactivation of the dACC (van der Wee et al., 2007; Koch et al., 2012), supplementary motor area (SMA), and inferior parietal lobule (Heinzel et al., 2018). Post-treatment, individuals with OCD are comparable to HCs in working memory load-dependent linear increases in cingulate activity, suggesting this modulation may be dependent on illness state rather than reflect broader underlying traits (van der Wee et al., 2007). Hypoactivation at higher cognitive loads may be explained by impairments in other executive functions (van der Wee et al., 2003; Heinzel et al., 2018), such as response monitoring and response inhibition, which are more often associated with these regions (Menzies et al., 2008; de Wit et al., 2012). For example, Heinzel et al. (2018) reported an association between lower response inhibition and hypoactivity in the SMA and the inferior parietal lobule during impaired working memory performance in individuals with OCD compared to HCs.

There are very few studies that have examined the neural correlates of working memory performance in AN and the inconsistent findings limit the overall patterns that can be drawn (see Table 2 for a summary of comparisons between AN and OCD). Biezonski et al. (2016) found alterations in thalamic morphology and functional connectivity between the thalamus, dorsolateral PFC, and anterior PFC that corresponded to behavioral deficits in working memory between individuals with AN and HCs. While others reported no differences in activation between adults with or without AN (Lao-Kaim et al., 2014). In contrast, others have reported neural correlates associated with enhanced working memory performance (e.g., Brooks et al., 2014; Israel et al., 2015). For example, hyperactivation of the dorsolateral PFC has been linked to better working memory performance in individuals with AN-R compared to individuals with AN-BP (Israel et al., 2015). In addition, greater dorsolateral PFC activity is associated with faster working memory performance and higher levels of obsessive-compulsive behaviors in adolescents with a diagnosis of Eating Disorder Not Otherwise Specified (EDNOS), an association that was not found in HCs (Brooks et al., 2014). Although not exclusively related to AN, these findings suggest efficient working memory performance in individuals with EDs characterized by AN-R type may be driven by ED-related ruminations that “train” the PFC through excessive use of working memory processes (Brooks et al., 2014). Potentially, individuals with AN-R may exhibit compensatory performance through other executive functions, such as response inhibition, which are less efficient in AN-BP and OCD. Further research is needed to directly compare the nature of ruminations and obsessions in OCD and AN and their associations to executive functioning performance.

In summary, cognitive and behavioral studies of working memory in AN and OCD have reported some overlapping findings, with reduced accuracy on visuospatial working memory tests present in both groups compared to HCs; however, these findings are more reliably found in OCD samples compared to AN samples. Research examining the neural correlates of working memory performance in both AN and OCD samples has implicated areas relevant to working memory processing and cognitive control, such as the dACC, dorsolateral PFC and temporo-parietal cortices. There is some evidence of hyperactivity of these regions in both groups, suggesting excessive cognitive control may coincide with impairments in working memory in both AN and OCD.

### Response Inhibition

Response inhibition is broadly defined as the ability to suppress a pre-potent behavior that is no longer required or appropriate and is a function of the broader executive function: *inhibitory control* (Chambers et al., 2009). Response inhibition can be differentiated into action restraint (measured using the Go/No-Go task) and action cancelation (measured using the Stop-signal task; van Velzen et al., 2014) and has been studied widely in relation to mental health conditions (e.g., Albrecht et al., 2005; Jahanshahi and Rothwell, 2017). Individuals with OCD display behavioral impairments in response inhibition compared to HCs, including increased commission errors on Go/NoGo tasks (Bannon et al., 2002; Penadés et al., 2007) and increased reaction times on Stop-Signal tasks (Menzies et al., 2007; Penadés et al., 2007; Boisseau et al., 2012; de Wit et al., 2012; Lei et al., 2015; McLaughlin et al., 2016). Response inhibition may represent a neurocognitive endophenotype of the disorder as similar impairments are present across unaffected first-degree relatives and individuals with OCD when compared to HCs (e.g., Menzies et al., 2007; Lennertz et al., 2012; Bora, 2020). Further support is provided by studies examining task-related neural activity pre-/post-treatment, with stable inhibition-related neural activity before and after exposure treatment reported in individuals with OCD (Thorsen et al., 2021). However, this literature is limited and inconsistent, as response inhibition performance has also been reported to improve in individuals with OCD following behavioral therapy (e.g., Nabeyama et al., 2008). Null results are reported in studies comparing response inhibition performance in individuals with OCD and HCs (Bohne et al., 2008; Tolin et al., 2014; Kalanthroff et al., 2016; Fan et al., 2017), but the consensus from multiple reviews and meta-analyses is that response inhibition deficits are a feature of OCD (Chamberlain et al., 2005; Lipszyc and Schachar, 2010; Abramovitch et al., 2013; Shin et al., 2014; van Velzen et al., 2014; Snyder et al., 2015; Norman et al., 2019). These reviews highlight the significant heterogeneity across studies which may be attributed to clinical factors, such as high co-occurrence with depression (Basso et al., 2001) and the heterogeneity of OCD (Hashimoto et al., 2011), as well as methodological factors, such as the specificity of the tasks for isolating different components of response inhibition and the impact of other executive functions on performance. Although not all studies find evidence of an association between response inhibition deficits and specific OCD symptoms (Lei et al., 2015), there is suggestion that response inhibition impairments predict severity of compulsions but not obsessions (Berlin and Lee, 2018), signifying a symptom-specific effect.

Children and adolescents with a diagnosis of OCD do not seem to follow the same pattern of poor response inhibition seen in adults. Research has consistently reported no differences in response inhibition performance between adolescents with OCD and HCs (e.g., Ornstein et al., 2010; Abramovitch et al., 2015; Negreiros et al., 2020; Wilton et al., 2020). It is worth noting that many studies in children with OCD diverge in the tasks utilized – using variations of the Stroop task that require not only response inhibition but also ignoring interfering information (i.e., set shifting) and response selection (i.e., working memory) – which have also produced null results (Andrés et al., 2007; Chang et al., 2007; Huyser et al., 2011; Lewin et al., 2014; Deepthi et al., 2021).

Compared to OCD, fewer studies have examined response inhibition in AN. Overall, studies either report impairments in response inhibition, including increased error rates and longer reaction times compared to HCs (Galimberti et al., 2012; Collantoni et al., 2016), or no differences between individuals with AN and HCs (e.g., Oberndorfer et al., 2011; Wierenga et al., 2014; Yue et al., 2020; Suttkus et al., 2021). Response inhibition impairments appear to be more pertinent for individuals with AN-BP eating behaviors (Galimberti et al., 2012), whilst adolescents with AN-R demonstrate enhanced response inhibition compared to HCs (Weinbach et al., 2020). Interestingly, this facilitation of response inhibition was specific to high-calorie food stimuli, suggesting this enhancement could serve as a maintenance factor for AN through enabling severe restriction of food intake. In contrast, food-related disinhibition could be related to increased impulsivity (Schag et al., 2013) and has been reported in individuals with AN-BP (Rosval et al., 2006), further supporting the argument that binge eating/purging behaviors are linked to response inhibition deficits. Impulsivity is thought to arise from inhibitory control deficits and may represent an important overlap between affect-regulatory binge-purge behaviors in AN-BP and compulsions in OCD (Altman and Shankman, 2009; Hoffman et al., 2012). Taken together, these findings highlight the limitation of assessing response inhibition in combined samples of individuals with AN-R and AN-BP subtypes and may help explain the null findings previously reported. Weinbach et al. (2020) also highlights the lack of studies examining response inhibition in adolescents with AN, who are more likely to experience shorter illness durations compared to adults and thus, may be less affected by deterioration of response inhibition abilities as the illness progresses. However, there is currently no support for this hypothesis and longitudinal studies assessing response inhibition that start from preadolescence are required.

The only study to-date that has directly compared individuals with OCD and individuals with an ED, has reported more pronounced deficits in the OCD group (Boisseau et al., 2012). They found individuals with OCD were significantly slower at inhibiting responses compared to HCs and more likely to make omission errors compared to individuals with an ED (Boisseau et al., 2012). It is important to note that the ED group were individuals who met criteria for an ED at a healthy BMI (limited to individuals with bulimia nervosa and EDNOS) to avoid the confound of low weight on neurocognitive outcomes and provide a heterogeneous sample of ED symptoms and diagnoses (Boisseau et al., 2012). This rare direct comparison suggests that there may be some differences in the etiology of binge eating/purging type EDs and OCD in the domain of response inhibition. Unlike set shifting and working memory where the evidence points to overlap in behavioral deficits with shared neural mechanisms, Boisseau et al. (2012) show that response inhibition may be more generalized for OCD but specific to food-related stimuli in binge/purge type EDs. While this limits the inferences that can be made to AN samples, it highlights the necessity for research to directly compare response inhibition abilities in OCD and AN, including distinguishing between AN-R and AN-BP.

Abnormalities in neurocircuitry known to mediate functions involved in response inhibition, such as the fronto-striatal circuit, have been consistently identified in OCD (e.g., Roth et al., 2007; Kang et al., 2013). Adults and children with OCD display hypoactivation of the dorsolateral PFC, inferior frontal gyrus, striatum, and thalamus during response inhibition on Go/NoGo and Stop-signal tasks compared to HCs (Roth et al., 2007; Woolley et al., 2008; Page et al., 2009; Rubia et al., 2010; de Wit et al., 2012). A meta-analysis combining structural and functional correlates of inhibition has extended these findings to demonstrate overlapping decreased gray matter volume and hypoactivation of the rostral and dorsal ACC and medial PFC, whilst increased gray matter volume and hyperactivation was reported in the left putamen and posterior insula in individuals with OCD relative to HCs (Norman et al., 2016). The authors suggest impaired inhibitory control in OCD may be due to enlarged and hyperfunctioning putamen and insula regions that are poorly regulated by underactive medial PFC and ACC regions (Norman et al., 2016). In addition, OCD symptom severity is reported to be inversely correlated with right OFC and ACC activation, suggestive of an involvement of these areas in suppression of symptoms; whereas, activation in thalamic and posterior cortical regions was positively correlated with symptom severity (Roth et al., 2007). Altered brain activation has also been observed in the pre-SMA during inhibition. Specifically, hyperactivation of the pre-SMA was reported by de Wit et al. (2012) in both individuals with OCD and their unaffected siblings compared to HCs during a Stop-Signal Task, and was related to better inhibitory task performance, leading researchers to suggest this activation could be compensatory.

Similar to OCD, neuroimaging studies with adolescents diagnosed with AN provide evidence of aberrant functioning in fronto-striatal regions during response inhibition (e.g., Lock et al., 2011; Wierenga et al., 2014; Bartholdy et al., 2019; see Table 2 for a summary of comparisons between AN and OCD). During successful inhibition responses, adolescents with binge eating and purging behaviors (e.g., AN-BP and bulimia nervosa), demonstrated increased activation of the right dorsolateral PFC and bilateral hypothalamus compared to both HCs and adolescents with AN-R (Lock et al., 2011). In addition, increased activation of the right ACC, bilateral precentral gyrus, and middle temporal gyrus was identified in adolescents with AN-BP and bulimia nervosa compared to HCs (Lock et al., 2011). These findings parallel the results from behavioral studies previously described, suggesting individuals with binge/purge behaviors demonstrate behavioral and neural correlates of decreased inhibitory control. Using a prospective longitudinal design, greater recruitment of medial prefrontal regions and the ACC during unsuccessful inhibition has also been found to contribute to the development of binge/purge behaviors in adolescence (Bartholdy et al., 2019). In addition, task demands appear to have a differential impact on neural activity during response inhibition in AN. As task difficulty increases, less inhibition-related activation has been reported in the dACC and the posterior cingulate cortex in adolescents with AN (Wierenga et al., 2014), and in the middle frontal gyrus in both adolescents with AN and adults who have recovered from AN, relative to HCs (Oberndorfer et al., 2011; Wierenga et al., 2014). The inferior frontal gyrus has also been implicated in impaired response inhibition in AN (e.g., Collantoni et al., 2016), which may suggest ventral attentional network disruptions are causing difficulties in adapting behavioral responses to the task demands in AN (Collantoni et al., 2016).

Electrophysiological studies examining the neural correlates of response inhibition focus on the N2 and P3 event-related potentials. An increase in N2 amplitude reflects detection of environmental inconsistency or conflict (e.g., NoGo trial) and the P3 commonly reflects increased evaluation of that stimulus. In OCD, studies report longer N2 latencies, as well as attenuation of the N2 amplitude, both are related to increased OCD symptoms (e.g., Herrmann et al., 2003; Kim et al., 2007). However, enhanced N2 amplitudes (Ruchsow et al., 2007) have been reported, as well as null results for N2 amplitudes (Herrmann et al., 2003) and P3 amplitudes between individuals with OCD and HCs (Herrmann et al., 2003; Kim et al., 2007; Ruchsow et al., 2007). To-date, only one electrophysiological study has examined the neural correlates of response inhibition in AN (Yue et al., 2020). In this study, individuals with AN displayed attenuated P3 amplitudes and delayed N2 latencies across three demand loads in a stop-signal task compared to HCs, suggesting delayed detection of conflict and impaired evaluation in AN (Yue et al., 2020).

Evidence from OCD suggests response inhibition impairments may represent a neurocognitive endophenotype of the disorder; whereas, research from AN suggests impairments are more nuanced and specific to individuals presenting with binge-purge symptoms. Although fronto-striatal circuitry has been consistently implicated in both groups during response inhibition, shared hypoactivation is specific to the ACC and frontal gyrus. Disorder-specific activity is observed in the dorsolateral PFC, with hypoactivation in OCD and hyperactivation in individuals with binge/purge type eating behaviors. This is an interesting distinction given the important role of the dorsolateral PFC in inhibition, and negative associations found between impulsivity and dorsolateral PFC activity in the general population (Asahi et al., 2004).

### Response Monitoring

Response monitoring involves monitoring one’s actions and their outcomes and adapting behavior to optimize performance (see Table 1). It is typically examined through conflict tasks in conjunction with other functions, such as response inhibition. Behavioral measures of response monitoring focus on post-error slowing, an indicator of increased response monitoring (Endrass and Ullsperger, 2014; see Table 1 for task examples and descriptions), as well overall performance indicated by accuracy and reaction time. Interestingly, the corpus of behavioral research on response monitoring in OCD suggests this executive function is unimpaired, however, the neuroimaging evidence, discussed below, may give more nuanced evidence. A small number of studies have identified increased task accuracy in individuals with OCD compared to HCs (e.g., Riesel et al., 2011, 2014). However, the majority found no differences in post-error slowing (e.g., Riesel et al., 2011, 2014; Hanna et al., 2012) or overall accuracy and reaction times (e.g., Gehring et al., 2000; Johannes et al., 2001; Nieuwenhuis et al., 2005; Endrass et al., 2008, 2010; Stern et al., 2010; O’Toole et al., 2012; Carrasco et al., 2013).

Compared to OCD, studies exploring the role of response monitoring in AN are mixed. A few studies report no differences in behavioral performance between individuals with AN and HCs (Wierenga et al., 2014; Geisler et al., 2017; Suttkus et al., 2021) and others providing some indicators of poorer (Ritschel et al., 2017) or better performance (Pieters et al., 2007) in individuals with AN. For example, compared to HCs on a flanker task, individuals with AN made fewer errors overall and were less likely to follow an error with another incorrect response (Pieters et al., 2007). However, HCs displayed more robust post-error slowing and individuals with AN tended to respond late following an error compared to HCs, which could indicate that the post-error responses were not always recorded (Pieters et al., 2007).

In comparison to behavioral findings suggesting no overt differences in response monitoring between individuals with OCD and HCs, electrophysiological studies provide strong evidence for excessive internalized response monitoring in OCD. Electroencephalography (EEG) is used to measure the error-related negativity (ERN) component, a negative ERP generated following incorrect responses on conflict tasks, such as flanker, Stroop, and Go/No-Go tasks (Endrass and Ullsperger, 2014; described in Table 1). Enhanced ERN amplitude in individuals with OCD was first reported in a seminal study by Gehring et al. (2000) using the Stroop task and has since been replicated across a range of conflict tasks in adults (Johannes et al., 2001; Nieuwenhuis et al., 2005; Endrass et al., 2008, 2010; Stern et al., 2010; Riesel et al., 2011) as well as children and adolescents with a diagnosis of OCD (Huyser et al., 2011; Hanna et al., 2012; Carrasco et al., 2013). Although, it should be noted that some studies fail to find a difference in ERN amplitude between individuals with OCD and HCs during errors on learning tasks (O’Toole et al., 2012; Doñamayor et al., 2014), suggesting this enhancement is specific to tasks emphasizing speed and accuracy. Neural correlates of enhanced response monitoring have also been reported in adults without a diagnosis of OCD who display high levels of obsessive-compulsive behaviors, compared to those who display low levels (Hajcak and Simons, 2002). In a community sample of children, a significant positive relation was found between ERN amplitudes and obsessive-compulsive behaviors (Santesso et al., 2006). This evidence, alongside reports of enhanced ERN amplitudes in individuals with OCD and their unaffected relatives compared to HCs (Carmi et al., 2019), suggests excessive response monitoring is associated with obsessive-compulsive behaviors independent of clinical diagnosis. Additional support for the state-independency of excessive response monitoring has been found in both adult and child OCD samples, with persistent enhanced ERN amplitudes post-treatment and symptom reduction (Hajcak et al., 2008; Huyser et al., 2011; Carrasco et al., 2013; Riesel et al., 2015).

Converging evidence from functional magnetic resonance imaging (fMRI), EEG source localization, and brain region research suggests the ERN is generated by the ACC (Dehaene et al., 1994; Kiehl et al., 2000; van Veen and Carter, 2002; Stemmer et al., 2004; Maltby et al., 2005; O’Connell et al., 2007). It is worth noting that compared to the other executive functions examined in this review, there are far less fMRI studies of response monitoring in OCD. The fMRI studies that have been conducted provide further support for enhanced response monitoring in OCD, as increased error-related activity in the ACC has been found in individuals with OCD compared to HCs. This elevated activity was also positively correlated to severity of their OCD symptoms (e.g., Ursu et al., 2003). Taken together, these findings provide strong support for excessive response monitoring in OCD across the lifespan. Further, evidence from electrophysiological studies provides a robust case for enhanced ERN amplitudes to represent a promising endophenotype for OCD.

To the best of our knowledge, there has only been one electrophysiological study to-date that has examined neural correlates of response monitoring in AN. This study reported attenuated ERN amplitudes compared to HCs during a Flanker task (Pieters et al., 2007). Unlike the other executive functions discussed in this review with substantial overlapping behavioral and neurological evidence between OCD and AN, these findings contrast with the majority of studies reporting enhanced ERN amplitudes in OCD. An explanation for the attenuated ERN amplitudes in AN could relate to dysfunction of the ACC. Individuals with AN present with reduced gray matter in the ACC, which is related to severity of the disorder as measured by BMI (Mühlau et al., 2007). fMRI studies have produced conflicting findings, as elevated ACC activity has been reported in one study with female adolescents who have a diagnosis of AN (Geisler et al., 2017), whilst others report no difference in ACC activity in individuals who have either recovered from AN or have a current diagnosis, compared to HCs (Wierenga et al., 2014; Ritschel et al., 2017; Suttkus et al., 2021). It is worth highlighting that none of these studies used a Flanker task in their designs, instead a probabilistic reversal learning task (Geisler et al., 2017; Ritschel et al., 2017), Stop-signal task (Wierenga et al., 2014), and Go/NoGo task (Suttkus et al., 2021) were employed. This is important as Riesel (2019) highlighted the importance of response conflict tasks in demonstrating altered ERN amplitudes in OCD in a recent meta-analysis. Therefore, tasks that do not include response conflict, such as probabilistic learning paradigms, may not require the same type of error processing as response conflict tasks, such as the Flanker task. Overall, the limited evidence to-date suggests that in AN there is attenuation of the ERN, but this may be specific to response-conflict tasks.

There are additional moderating factors that should be considered when comparing response monitoring performance in AN and OCD. For example, higher levels of response monitoring and elevated ERN amplitudes are associated with increased perfectionism (Perrone-McGovern et al., 2015; Stahl et al., 2015; Barke et al., 2017) and intolerance of uncertainty (Jackson et al., 2016). Individuals with maladaptive perfectionism (high concern but low standards) display larger ERNs compared to adaptive perfectionists (high concern and high standards) and non-perfectionists, which may reflect a decreased capacity to regulate anxiety after committing an error relative to adaptive perfectionists (Perrone-McGovern et al., 2015; Barke et al., 2017). Intolerance of uncertainty is broadly defined as the tendency to hold negative beliefs about uncertainty and react negatively to uncertain situations and events, even if the potential threat is small (Brown et al., 2017). Hyperactivation of the amygdala, ACC, as well as frontal areas, such as the dorsolateral PFC and OFC, all indicative of excessive cognitive control, are reported to be related to intolerance of uncertainty in OCD (Wever et al., 2015).

Levels of anxiety are also important to consider when examining ERN amplitudes. For example, studies carried out to explore error-related brain activity are conducted in laboratories by researchers and can create a clinical and unnatural environment, which is amplified when the individual is asked to participate in tasks monitoring their performance. Task features such as trial-by-trial feedback could increase levels of anxiety and potentially exacerbate obsessive-compulsive behaviors by providing a salient negative cue to the individual, highlighting their mistake on error trials. On the other hand, trial-by-trial feedback could serve to decrease anxiety by offering reassurance to the individual, providing an external evaluation of their performance, and hence reducing the burden on their own response monitoring (Nieuwenhuis et al., 2005; Doñamayor et al., 2014). Therefore, both state and trait anxiety are useful measures when interpreting findings from studies exploring ERN amplitudes, especially in light of the strong relationship between anxiety and OCD (Bartz and Hollander, 2006).

In summary, response monitoring may help distinguish between AN and OCD, as there is neuroimaging evidence of excessive response monitoring in OCD, whilst evidence from AN suggests there is attenuation of the ERN (see Table 2 for a summary). Both disorders present with preserved task performance compared to HCs. Differences in ERN amplitudes between AN and OCD could relate to differences in ACC function, although ACC function in AN may be yoked to malnutrition. The paucity of evidence reporting neural and behavioral response monitoring in AN and the lack of sensitivity to the distribution of AN subtypes, participant gender, and co-occurring anxiety in these studies limit the direct comparisons that can be made. Further evidence is needed in weight-recovered AN patients and prospective longitudinal studies starting before the ED develops to disentangle these factors.

## Conclusion and Future Directions

Overall, findings from this review suggest neural mechanisms underlying executive functioning in AN and OCD appear to diverge from shared behavioral markers (summarized in Table 2). There are three key conclusions from this review. The first is the importance of examining both behavioral and neural markers when studying transdiagnostic correlates of executive functions. Based on behavioral evidence only, AN and OCD appear to share broad overlapping impairments in executive functioning. However, neurological evidence demonstrates a divergence in the neural correlates of executive functions in AN and OCD. Second, is the possibility that the divergence in neural correlates may reflect different compensatory mechanisms in each disorder. Neural structures and networks may act differently in AN and OCD to compensate for difficulty in areas of executive function, even though they both present with behavioral impairments (comparisons are summarized in Table 2). Thirdly, there may be multiple neural pathways that subserve each executive function and individuals with AN and OCD may utilize different neural pathways.

This review also highlights evidence of shared traits that may be linked to the presentation of executive functioning in AN (including AN subtypes) and OCD. For example, increased perfectionism has been associated with better set shifting performance in AN (Lindner et al., 2014; Vall and Wade, 2015); however, this enhancement may be specific to AN-R rather than AN-BP (e.g., Herbrich et al., 2018). In turn, this may suggest impairments in set shifting performance are more characteristic of OCD (e.g., Bohon et al., 2020) and potentially AN-BP. Although further research in AN-BP samples is needed to support this hypothesis. In addition, impulsivity is associated with response inhibition deficits (e.g., Chambers et al., 2009) and is a feature of both OCD and AN-BP, but not AN-R (Altman and Shankman, 2009). Response inhibition deficits appear to be more pertinent in individuals with OCD (e.g., Penadés et al., 2007) and AN-BP (e.g., Galimberti et al., 2012), whilst individuals with AN-R subtype demonstrate enhanced response inhibition (e.g., Weinbach et al., 2020). This again suggests a specific overlap in cognitive impairments and shared traits between AN-BP and OCD, compared to AN-R. It also highlights the need for future research to sample for specific AN subtypes when examining executive function.

Another consideration for future research is the current inconsistency in measures used to examine executive functions across AN and OCD samples. For example, set shifting performance is measured across a range of tasks, including the WCST (Heaton and Staff, 1993) which captures “attentional” set shifting, the Haptic Illusion task that measures “perceptual” set shifting (e.g., Tchanturia et al., 2002), and the Object Alternation Test (OAT; Freedman, 1990) which captures “affective” set shifting (Deepthi et al., 2021). These types of set shifting may also be mediated by different brain areas, such as the OFC for affective set shifting (Remijnse et al., 2006), and dorsolateral PFC and ventrolateral PFC for attentional set shifting (Nagahama et al., 2001). Even considering findings from the WCST alone, studies differ in the outcome measures used, such as the number of categories completed (Kashyap et al., 2013), total errors (Wilsdon and Wade, 2006), perseverative responses (Van Autreve et al., 2013), and non-perseverative errors (Tchanturia et al., 2012). Response inhibition impairments in AN and OCD may also be specific to the type of inhibition studied. For example, this review focused specifically on response inhibition, but interference control has also been examined in OCD, and appears to depend on overlapping but distinct, brain areas compared to response inhibition (see van Velzen et al., 2014 for a comprehensive review).

Weight status, specifically related to low body weight and malnutrition, is important to consider when examining data from AN samples. For example, less robust evidence of executive functioning impairments in children and adolescents with AN has been interpreted as more subtle cognitive disturbances that are exacerbated by long term starvation and maintain the illness in adults with AN (Lang et al., 2014). This may also impact on neural activation differences observed between children/adolescents and adults with AN (Lao-Kaim et al., 2014). In addition, although neuroimaging evidence to-date suggests differences in ERN amplitudes between AN and OCD could relate to differences in ACC function, ACC function in AN may be yoked to malnutrition (e.g., Mühlau et al., 2007). It is therefore important to consider what impact weight restoration has on cognitive functioning in individuals with AN (i.e., are there improvements in previously impaired functions post-weight recovery?). Neurological scar effects, defined as neuropsychological features that can be exacerbated by previous prolonged illness and may persist after weight recovery, are also key to future examinations of cognition in AN (Lang et al., 2014). It may also be the case that transdiagnostic factors present in both AN and OCD that are linked to overlapping cognitive impairments may be masked by the impact of starvation and malnutrition on the brain in AN.

Co-occurring conditions, medication, and IQ are commonly controlled for in studies examining executive functioning in AN and OCD samples. However, studies vary in the exclusion criteria they employ. For example, studies examining set shifting performance in OCD typically control for verbal IQ and exclude participants who have co-occurring neurological conditions or psychiatric diagnoses (e.g., Okasha et al., 2000; Rajender et al., 2011). However, a large number of studies did not exclude participants based on a co-occurring diagnosis and/or did not measure levels of anxiety and depression (e.g., Aronowitz et al., 1994; Bohon et al., 2020). Many studies did not directly control for medication (e.g., Veale et al., 1996; Lawrence et al., 2006; Chamberlain et al., 2007); however, studies that did reported no effect of medication on behavioral or neural measures (Andrés et al., 2007; Vaghi et al., 2017; Geller et al., 2018). There have been inconsistent findings concerning the impact of co-occurring clinical symptoms, such as depression, on executive functioning performance. Some researchers have argued that depression accounts for executive functioning impairments in individuals with OCD (e.g., Basso et al., 2001; Aycicegi et al., 2003), whereas others report no impact of co-occurring depression on performance (e.g., Abramovitch et al., 2011; Snyder et al., 2015; Geller et al., 2018). In AN, co-occurring depression symptoms are also argued to play a significant mediating role in executive functioning impairments; however, they do not completely account for these deficits (Giel et al., 2012; Weider et al., 2015). Therefore, future studies examining executive functioning in AN and OCD samples should ensure a control group is matched on age, sex distribution, and verbal IQ, and should either exclude participants based on medication use and co-occurring diagnoses or ensure these are adequately measured and controlled for in analyses.

There is also the possibility that additional executive functioning abilities can impact on performance in executive functioning tasks. For example, greater working memory has been associated with larger ERN amplitudes relative to weaker working memory, as well as better attentional control and academic performance in the general population (Weinberg et al., 2015). Response inhibition has also been found to compensate for set shifting difficulties in individuals with AN (Weinbach et al., 2019). This suggests response inhibition deficits may be driving the impairments in set shifting, especially for studies employing set shifting measures that require both inhibition and set shifting ability. This is important to consider, as difficulties in response inhibition and set shifting appear to be more characteristic of OCD (e.g., Boisseau et al., 2012; Bohon et al., 2020) and AN-BP (e.g., Galimberti et al., 2012), but enhancement of these functions is seen in AN-R (e.g., Herbrich et al., 2018; Weinbach et al., 2020). Therefore, controlling for other aspects of executive functioning may be important for disentangling specific difficulties presented by individuals with AN and OCD.

To investigate the shared underlying mechanisms between AN and OCD, future research should consider the symptoms present in both disorders dimensionally, in line with the NIMH’s Research Domain Criteria (RDoC; Insel et al., 2010). This will better capture and identify overlapping symptoms present in both AN and OCD (e.g., ruminations), as well as their relations to cognitive functioning. For example, compensatory repetitive behaviors present in individuals with OCD (e.g., checking behavior) and AN-BP (e.g., purging behavior) may be driven by impairments in set shifting and response inhibition, whereas, in individuals with AN-R, enhanced set shifting may facilitate restrictive eating behaviors. Future research should probe these executive functions further in individuals with OCD, to examine OCD subtypes. In OCD, it could be predicted that people who show high levels of compulsive rituals may be particularly expected to show set shifting and response inhibition impairments, as compulsions relate to checking behaviors, which have previously been related to set shifting and response inhibition impairments (Leopold and Backenstrass, 2015; Berlin and Lee, 2018). These findings could provide support for a transdiagnostic association between compulsions and impaired set shifting and response inhibition performance. Indeed, there is mounting evidence for a transdiagnostic approach to conceptualizing mental health problems due to its potential for novel approaches to treatment, research, and our understanding of the etiology of mental health problems (e.g., Dalgleish et al., 2020). This extends the state of knowledge currently accessible through categorical approaches, which can be limited when we consider the multifaceted and often complex nature of mental health problems, where the causes are still poorly understood.

Very few studies have prospectively examined associations between neural correlates of executive functioning in childhood and later ED and OCD symptoms, suggesting this is another important future research direction. A comprehensive review by Culbert et al. (2015) endorsed a clear need for longitudinal research in EDs examining high-risk samples, such as those with elevated disordered eating, advising for these studies to start in preadolescence to identify antecedents and capture risk trajectories of eating pathology. In addition, individuals with a neurodevelopmental condition, such as autism spectrum disorder (ASD) or attention deficit hyperactivity disorder (ADHD), may represent a clear “at-risk” group for the development of AN and OCD, as studies commonly report executive functioning impairments in these samples (e.g., Ozonoff and Jensen, 1999; Lawson et al., 2015; Craig et al., 2016). There is also a considerable amount of research documenting the overlap between ASD and both AN and OCD, as well as shared impairments in executive functioning performance (e.g., Delorme et al., 2007; Zandt et al., 2009; Westwood et al., 2016; Zhou et al., 2018). Further, ADHD in childhood has been shown to increase the risk of later developing OCD (e.g., Geller et al., 2007) or an ED (e.g., Biederman et al., 2007). Taken together, these points provide some promising avenues for future research to develop our understanding of the similarities observed between AN and OCD, as well as potential shared mechanisms.

The findings presented in this review also have important implications for treatment. In people with AN, the presentation of obsessive-compulsive features alongside ED symptoms can lead to a more severe and enduring illness (Milos et al., 2002; Cumella et al., 2007). This suggests obsessive-compulsive features facilitate the maintenance of the ED, possibly leading to these cognitions and behaviors becoming more entrenched over time. Based on this review, an explanation for this could be that difficulties in executive functioning lead to impairments in behavior adaptation and cognitive flexibility that facilitate an increase in obsessive and compulsive behaviors, supporting AN symptom maintenance. As the AN progresses, these cognitive functions become more impaired over time, leading to an increasingly rigid cognitive style and more obsessive and compulsive behaviors, with increasingly entrenched ED cognitions. Promising research by Lee et al. (2020) found that targeting cognitive patterns and specific OCD symptom dimensions, such as obsessing, neutralizing, and ordering, in addition to ED-focused treatment resulted in enhanced treatment outcomes for women/men with an ED. Furthermore, treatment options that are individualized and targeted at addressing additional overlapping traits, such as perfectionism and intolerance of uncertainty, as well as internalizing symptoms, may be beneficial for individuals with AN. For individuals with OCD, co-occurring disordered eating behaviors may also have implications for treatment outcomes. For example, lifetime suicide attempts, as well as levels of anxiety and depression, are found to be elevated in individuals with a primary diagnosis of OCD and subsequent co-occurring ED diagnosis, compared to individuals with an OCD diagnosis only (Sallet et al., 2009). The presence of a co-occurring AN diagnosis in individuals with OCD may therefore lead to more severe clinical characteristics and a more complex AN/OCD presentation. It is also important to consider the impact of malnutrition in individuals with co-occurring AN and OCD. For some individuals, the primary goal may be weight restoration due to physical health deterioration and the decreased ability to participate in cognitive therapies, such as cognitive behavioral therapy and exposure and response prevention (Lewin et al., 2013). Anxiety may become more intense as weight restoration progresses, and alongside a delay in engagement with cognitive therapies, may result in more severe and enduring OCD symptoms.

Shared behavioral impairments may also have broader implications for treatment of both AN and OCD, such as cognitive training targeting specific cognitive impairments, including executive functions. Cognitive remediation therapy (CRT) is an intervention aimed at improving cognitive flexibility, information processing, reducing perfectionism, and awareness of maladaptive thinking styles (van Passel et al., 2016). It has provided promising results in AN samples, with improvements in cognitive performance reported (Tchanturia et al., 2014, 2017). However, a recent randomized controlled trial comparing the effectiveness of CRT and a control treatment as add-ons to treatment as usual found that CRT did not enhance the treatment as usual more than the control treatment in both AN and OCD samples (van Passel et al., 2020). Important to note is that CRT is primarily delivered verbally by a trained mental health professional, but research suggests computerized neurocognitive training may provide a better treatment response due to the increased frequency and duration of practice possible (for a review of cognitive training in EDs, see Juarascio et al., 2015). In addition to CRT, cognitive training focusing on organizational strategies and problem solving has been studied in individuals with OCD, producing improved memory function and alleviation of clinical symptoms in individuals who received the intervention compared to individuals with OCD who did not (Park et al., 2006). Early intervention is key to improving treatment outcomes in both disorders (Treasure and Russell, 2011; Fitzgerald et al., 2021) and cognitive training targeting executive functioning impairments during the early stages of AN and OCD may provide a particularly promising avenue for future research. Further, employing preventative cognitive training before the disorder develops may help protect against its development. For example, cognitive training for at-risk individuals who experience high levels of disordered eating and/or obsessive-compulsive behaviors alongside executive functioning difficulties, may help protect against increased severity of these behaviors and the development of AN and OCD. Lastly, it is important to consider the context in which executive functions are measured. Contextual factors related to medication, psychological/behavioral therapies, and severity of illness should be considered. For example, some studies have found enduring executive functioning impairments in adults with AN following weight-restoration (Roberts et al., 2010; Danner et al., 2012), whilst improvements in these functions have been found in younger age groups (Castro-Fornieles et al., 2010). Although not directly comparable to recovery in the form of weight restoration in AN, medication has been found to be effective at treating OCD despite enduring executive functioning deficits (Kim et al., 2002; Nielen and Den Boer, 2003). This suggests different treatments may have different mechanisms for change in AN and OCD. Improving executive functioning through cognitive training is one approach that can support recovery and potentially compliment or enhance current treatment approaches.

This review has provided a synthesis of the existing research on executive functioning in AN and OCD in the context of broader trait profiles. We have highlighted the importance of future research examining both behavioral and neural markers when studying transdiagnostic correlates of executive function. In addition, findings from the review suggest the possibility of multiple neural pathways subserving executive functions and alternative compensatory mechanisms utilized in each disorder. Future research should examine direct links between neuroimaging findings and disorder-specific behaviors to help infer brain-behavior associations. Furthermore, studies should consider using a transdiagnostic approach to conceptualize mental health issues and longitudinal research designs that begin in younger age groups. This will help identify antecedents and capture risk trajectories of both eating pathology and obsessive–compulsive symptoms.

## Author Contributions

KT and RB were responsible for collating and summarizing relevant literature. KT led on drafting the manuscript. RV and CJ revised for important intellectual content. All authors were involved in the conceptualization and design of the review, viewed the manuscript, and gave their approval before submission.

## Conflict of Interest

The authors declare that the research was conducted in the absence of any commercial or financial relationships that could be construed as a potential conflict of interest.

## Publisher’s Note

All claims expressed in this article are solely those of the authors and do not necessarily represent those of their affiliated organizations, or those of the publisher, the editors and the reviewers. Any product that may be evaluated in this article, or claim that may be made by its manufacturer, is not guaranteed or endorsed by the publisher.
